# Strengthening teamwork and respect (STAR) in maternity units: developing a health system intervention in South Africa

**DOI:** 10.1080/16549716.2024.2440982

**Published:** 2025-02-03

**Authors:** Tanya Doherty, Ruwayda Petrus, Sandra Land, Christiane Horwood, Veronique Filippi, Lyn Haskins, Cleo Phewa, Sphindile Mapumulo, Silondile Luthuli, Vaughn M. John

**Affiliations:** a Health Systems Research Unit, South African Medical Research Council, Cape Town, South Africa; b School of Public Health, University of the Western Cape, Cape Town, South Africa; c Department of Paediatrics and Child Health, University of Cape Town, Cape Town, South Africa; d Discipline of Psychology, School of Applied Human Sciences, University of KwaZulu-Natal, Durban, South Africa; e Adult and Community Education Unit, Durban University of Technology, Durban, South Africa; f Centre for Rural Health, University of KwaZulu-Natal, Durban, South Africa; g Department of Infectious Disease Epidemiology and International Health, London School of Hygiene and Tropical Medicine, London, UK; h School of Education, University of KwaZulu-Natal, Pietermaritzburg, South Africa

**Keywords:** Maternity care, respectful care, teamwork, health system intervention, participatory learning and action, learning health systems

## Abstract

Disrespect and abuse in maternity services in South Africa has been described over several decades and are rooted in the country’s complex socio-political landscape and unequal health system which places strain on public sector health professionals. Strategies to improve the quality of health care typically involve once-off didactic teaching or outside technical consultants focused on improving specific health programmes. These approaches fail to encourage self-reflection or to establish learning cultures. Participatory learning processes, embedded in routine service delivery, are a potentially powerful way to improve ownership and accountability for health system performance. We describe the process followed to develop the Strengthening Teamwork and Respect (STAR) intervention which is being implemented in nine district hospitals in two rural districts of KwaZulu-Natal. The intervention approach draws on a conceptual framework for learning health systems, with intervention strategies informed by participatory learning and action theory. The intervention design was an iterative process informed by literature reviews, formative data collection, consultation with provincial, district and hospital management stakeholders, expert reviewer inputs and piloting of proposed activities. This process produced the STAR intervention approach and toolkit, consisting of: identification and training of champions, creation of STAR teams, convening of learning sessions to work through STAR toolkit activities, identification, implementation and monitoring of change projects, and onsite and virtual mentorship from the STAR development team. Endline cross-sectional surveys and a parallel process evaluation will advance the evidence base for interventions to improve respectful care and cultures of teamwork and learning within maternity units in rural low- and middle-income settings.

## Paper context

- **Main Findings:** Grounded in a recognition of health systems as learning organisations and the need for sustainable learning and change processes to be locally driven, we brought together a multi-disciplinary team to design the STAR intervention to improve respectful maternal and newborn care.
- **Added Knowledge:** The process involved in developing public health interventions often receives less attention and funding than the evaluation of such interventions and has been referred to as the ‘black box’ of intervention research.
- **Global health impact for policy and action:** There is value in documenting the development of complex public health interventions to expose the ‘black box’ and to enable other researchers to share in the learnings.

## Background

Disrespect and abuse in maternity services in South Africa (SA) has been well described over several decades [1–4]. Such experiences have been found to be rooted in the country’s complex socio-political landscape and unequal health system, placing strain on health professionals in the public sector [1,5]. Rural districts in SA face particular challenges related to attracting and retaining skilled health professionals [6]. These challenges have a profound effect on the quality of patient care and health outcomes.

As in most other low- and middle-income countries (LMICs), decentralised governance in SA has led to increased responsibility for service delivery at the level of districts, often without efforts to strengthen management capacity, develop teamwork or a culture of continuous learning [7,8]. Rural health professionals are faced with the challenge of trying to provide good patient care in particularly resource-constrained contexts [9,10]. Distances from specialist care mean that health professionals in rural maternity units face rapidly developing emergency situations threatening the lives of women and babies, without adequate resources [11]. Health professionals in these contexts can experience powerlessness, apathy and burnout [10,12].

The transformation of organizations into learning organizations has been proposed as a key strategy for improving their effectiveness and efficiency [7,13]. For the health care sector, the ability to learn and to do so in a self-directed and ongoing manner, is essential since knowledge and skills can rapidly become obsolete and policy and guidelines change continuously [7,14]. This is crucial for health professionals’ job satisfaction, professional development and the overall quality of health care [14,15]. Continuous professional development (CPD) is thus a requirement for some levels of health professionals in SA, such as doctors, psychologists and social workers, and is likely to be extended to nurses and midwives in the future.

Participatory learning processes, embedded in routine service delivery, are a potentially powerful way to improve ownership and accountability for health system performance and quality of care [16,17]. In LMICs, various participatory approaches have been applied within maternal and newborn care, with different degrees of participation, such as maternal and perinatal death audits, quality improvement cycles and women’s groups practicing participatory learning and action (PLA) [18]. PLA is a family of approaches and methods which enable and empower people to share, analyze and enhance their knowledge of their life and conditions, and to engage in cycles of plan, act, monitor, evaluate and reflect [19]. The main purpose of PLA is to support people within communities of interest to analyse their own situation, rather than have it analysed by outsiders, and to ensure that any learning is then translated into action [20]. PLA groups are intended to be self-organized and members are encouraged to engage with each other regularly to solve problems. In the case of health professionals, the learning is focussed on improving the organizational environment and practice. A literature review of approaches for developing leadership and management competencies in LMIC health systems pointed to the potential that PLA approaches have to improve management and leadership within health teams at district and facility level [21]. In the field of maternal and newborn health, PLA approaches are particularly relevant due to the requirement to have multi-disciplinary teams comprising midwives, neonatal nurses, obstetricians, paediatricians, neonatologists, breastfeeding counsellors, home visitors, social workers and others. A further advantage is that the visual and participatory methods used in PLA help to level the learning space when groups with diverse educational and literacy levels are brought together [19].

However, the PLA approach is not without its challenges. While there is value in bringing together diverse members of teams, bringing together non-peer groups, into the deliberative processes has the potential to affect the impact and outcomes if issues of power sharing, hierarchy and participatory involvement are not addressed at the outset [22,23].

Team learning meetings and other approaches to encourage reflective practice have been used within the health system, for example, clinical audits. However, these have not always been found to lead to actions that improve quality of care due to a range of factors including cultures of blame, inability to demonstrate progress, lack of good facilitators and lack of accountability mechanisms [24]. Published quality improvement approaches also commonly use routine health information as their starting point for understanding problems assuming that quantifiable indicators (e.g. the proportion of women attending antenatal care) are readily interpreted and can trigger upwards accountability [25,26]. In line with the transformative paradigm, this study sought to gain insight into the research problem by both relying on existing data and engaging stakeholders within the health system in the development of the intervention approach [27]. This paradigm valorises the knowledge of health professionals and their agency to transform their workspaces and relationships. In this paper, we describe the process followed to design the strengthening teamwork and respect (STAR) intervention.

## Methods

### Participants and setting

**Figure 1.**
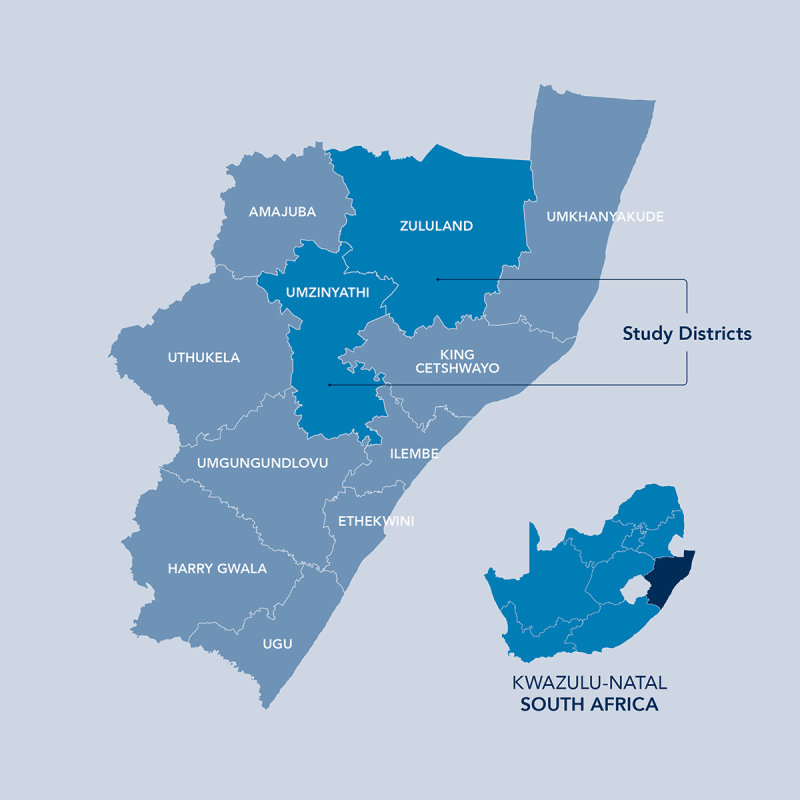
Map showing the study districts.

The target population for this project was health professionals in maternity units in two rural districts (uMzinyathi and Zululand) in KwaZulu-Natal, South Africa (Figure 1). These two districts were chosen because of their high maternal mortality levels (75 deaths per 100 000 live births in Zululand and 73 deaths per 100 000 live births in uMzinyathi) [28,29], low population density, geographical remoteness and long travel distances between district and secondary level referral hospitals.

uMzinyathi is a deep rural and mountainous district with poor basic infrastructure, little economic growth and a low population density at 64 people per square kilometre. Sixty-six percent of the population lives below the lower poverty line [28]. Zululand is the biggest district in Kwazulu-Natal making up 16% of the geographical area. It is a rural district with half of the area under the jurisdiction of traditional authorities. The population density is 60 people per square kilometre and 70% of the population live below the lower limit poverty line with two municipalities regarded as the poorest in the country [29].

All pregnant and lactating women and children under the age of six receive free healthcare in the public sector. Community-based primary health care is undertaken by ward-based outreach teams of community health workers through household visits [30,31]. Women with low-risk pregnancies receive antenatal care at primary care clinics and community health centres [5]. District hospitals provide level 1 (generalist) services including obstetric care for women with low-risk pregnancies. In total, there are nine district hospitals in the two districts where low risk vaginal deliveries are conducted by midwives and advanced midwives as well as low risk elective and emergency caesarean deliveries by generalist doctors (medical officers). The number of public sector medical practitioners per uninsured population is amongst the lowest in the country at 14.8 per 100,000 for Zululand and 17.6 per 100,000 for uMzinyathi in 2020 compared to a national average of 33.6 [32].

### Formative research to inform the STAR intervention implementation design

We used complementary mixed-method approaches during a formative phase to inform the content of the toolkit and training. See Supplementary Figure S1 for a schematic of the formative research methods. Briefly, the formative research consisted of two quantitative surveys: (1) The person-centered maternity care (PCMC) survey undertaken amongst women aged 16–49 years who had given birth in the previous 9 weeks. Full details of the methods and findings from this survey are available elsewhere [33]. (2) The Dimensions of Learning Organisation Questionnaire (DLOQ) administered to maternity unit staff to measure organisational learning culture within the health facilities [34]. The qualitative component consisted of ten focus group discussions (FGDs) with women who had given birth in participating facilities in the previous 9 weeks, five of which comprised women whose infants had been admitted to the neonatal units. The purpose of these discussions was to understand women’s perceptions of service availability, acceptability and their recent pregnancy and postnatal care experiences. This number of groups was deemed sufficient to enable a mix of small and larger hospitals across the two districts.

In-depth interviews were also conducted with a sample of health professionals and unit managers in the participating maternity units to explore existing learning processes in their units, both formal and informal, as well as their experiences, aspirations and local constraints and parameters for care giving. We chose to utilize focus group discussions for recipients of care because they enabled women from the same community to share their childbirth experiences with the potential that they could provide support to each other which could aid in their recovery process and emotional well-being for those who had negative experiences of childbirth. For health professionals, we chose individual interviews as we included staff from different professions and levels of seniority within the hospitals. We believed that group discussions within a hierarchical structure may have led to junior nurses being less able to share their perspectives compared to managers or doctors.

The two surveys were triangulated with the qualitative data to form a baseline assessment of the maternity service context, especially how and why rural maternity units function as they do, to understand the main bottlenecks to effective organizational learning culture and person-centered care.

### Intervention development process

Through funding from a research grant, we brought together a multi-disciplinary team to develop the STAR intervention, acknowledging the need for skills in organisational psychology and adult education as well as more traditional public health disciplines. Following a search of the literature for intervention approaches to improve teamwork and respectful care in the context of maternity units in low- and middle-income countries, we decided to align the implementation approach to the theory of learning health systems which underscores that health systems are complex, adaptive and people-centered with continuous learning being fundamental to improving health system quality [35]. We chose the Menear et al. conceptual framework for learning health systems [36] to guide our intervention development since it advocates for communities of interest (e.g. maternity unit teams) to come together to clarify problems, reflect, make decisions and effect change, through learning and improvement, with the ultimate aim for this approach to be embedded within health care facilities in order to strengthen health care teams and improve maternity care. The Menear et al. [36] framework was developed in Canada but the concept of ‘learning health systems’ as ecosystems of change is now increasingly used globally to describe approaches that encourage critical reflection, action and learning to improve quality of care and health outcomes [7,13,35]. In 2021, the WHO published a flagship report on learning health systems which recognizes that team and participatory approaches are important for optimizing learning capacity within health systems [35].

An important component of learning health systems is participation of staff in identifying challenges and working towards a common goal. We chose to apply a PLA approach to foster a culture of organisational learning. PLA is one approach amongst a wide range of methods for supporting organisational learning [37]. We chose this approach because it draws on peer learning and local knowledge and experience, making it suited to resource constrained as well as better-resourced health system contexts [37]. Through the choice of the PLA approach, we sought to create an enabling environment that would facilitate a problem-solving approach to improve health professionals’ skills to identify and solve challenges as inter-professional teams and act as change agents to improve care for women, newborns and their families.

### Provincial and district management engagement

In order to align the STAR intervention with the needs of provincial, district and hospital management and to ensure the design would be suitable for embedding within routine existing learning activities, the study team engaged with these stakeholders to present the findings from the formative research and to discuss the approach to strengthen maternity teams to provide respectful care. At the provincial level the study team met with the provincial heads of obstetrics, neonatal nursing and maternal and newborn health in a one-day workshop at the provincial department of health. For district stakeholders the STAR development team attended one of the regular monthly district health management meetings and at the hospital level a meeting was held with the hospital manager, clinical manager and maternity unit manager. At all of these engagements, open discussions were held where stakeholders were able to share their priorities and preferred approaches for addressing the intervention focus as well as their thoughts on how to sustain teams following completion of the research and approaches to scale up the approach to other districts. Such consultations informed the use of the terms ‘quality improvement’ rather than participatory learning and action in the intervention toolkit as this would align better with existing language used in in-service learning activities and would increase the likelihood of the project being embedded in routine quality improvement activities. These discussions also assisted with understanding existing interventions and priorities within the province.

During this period of engagement, the STAR development team also arranged informal, overt observations of existing learning activities within maternity units and noted the ways in which these sessions were facilitated, how staff participated, what content that was covered, what venues were used for learning, and the duration of learning activities. These observations provided useful insights into current team dynamics and organisational culture of learning and how the STAR intervention could best be integrated into existing learning activities in the hospitals. For example, such observations guided the intervention development in terms of a suitable duration for proposed STAR learning sessions and what resources would be available to STAR teams in their learning. The STAR development team reassured health professionals that they were not present to evaluate how learning sessions were being conducted but to observe and understand what a typical learning session at the facility may involve.

### The STAR toolkit

**Figure 2.**
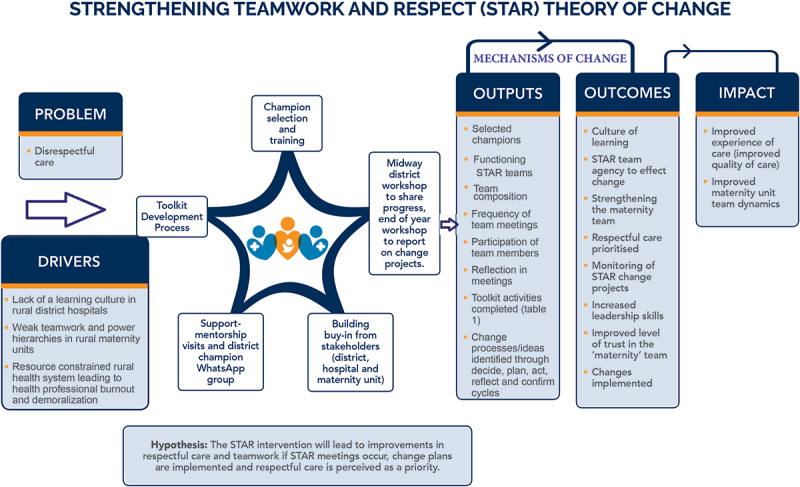
STAR theory of change.

We developed a theory of change to guide the development of intervention inputs and anticipated outputs and outcomes (Figure 2). Drawing on the formative research findings regarding differences between health professionals in opportunities for learning and women’s experiences of differing quality of care provided by different health professionals, the STAR intervention purposively sought to create multi-disciplinary teams within each maternity unit led by a facility-proposed champion and deputy champion with the aim of following a decide, plan, act, reflect and confirm cycle across learning sessions to effect change. We chose to use the term ‘champion’ because this term has been used in health systems interventions to reflect someone who is internal to an organisation with an interest and commitment to implementing change [38].

The decision was made that the main product to guide the implementation of learning sessions within maternity units would be a toolkit with structured participatory activities (Table 1) related to the priorities and gaps identified in the formative phase and from stakeholder consultations. For example, the first activity involved a daily schedule exercise to explore roles and responsibilities of the different group members and to discuss the most feasible time to hold group meetings as finding a time with minimal disruption to clinical duties was deemed important by district and hospital management. Vignettes (supplementary file), graphic representations of clinical scenarios (supplementary file), an animation (supplementary file), videos and role plays of both health professionals’ and women’s experiences drawn from the qualitative formative research were included in activities. The idea to create an animation video from the data collected as part of the formative phase, emerged during the preparation phase for the intervention. Part of the toolkit development involved reviewing a variety of resources from different regions around the world. While these resources were valuable in guiding the development of the toolkit, the STAR development team recognised the need for content was that directly informed by the insights gathered from the focus group discussions with mothers, specifically their lived experiences. This led to the decision to develop an animation video paired with engaging activities (such as role plays) as more localized and relevant formats for the toolkit. The findings from the PCMC survey [33] highlighting poor communication between health professionals and women led to a focus on activities that encourage health professionals to reflect on how women experience care and to role-play common scenarios in maternity care. The order of activities was guided by recommendations from the adult education experts such that activities to build trust within the STAR teams were undertaken with sufficient time allocated before the activities focused on the topic of respectful care.

**Table 1.** Activities in the STAR toolkit (teams have freedom to choose the order in which to undertake activities).

Activity	Aim
1. Scheduling STAR learning sessions	Orientate team to the manual and daily activity chart to identify suitable days and times for the team to meet.
2. Reflection on being a health care professional as a vocation: silent graffiti	To facilitate health professionals’ reflection on health work as their career, and encourage expression of own opinion.
3. Gathering ideas for change projects	To provide everyone with a chance to share their ideas and dreams for change in the maternity unit.
4. Walk in the footsteps of your patients	To explore familiar health facility surroundings with a critical eye and develop critical awareness.
5. Ranking ideas for change projects	For the STAR team to select a few change projects by gathering all the ideas from the above activities, ranking them, and identifying their top 3 project ideas.
6. Patients’ perspective: what would they say?	To consider situations from a patient’s point of view – an exercise in respectful care using illustrations of maternity unit scenes with blank thought and speech bubbles.
7. Roleplays: Situations where it may be difficult to give respectful care	To role play scenarios involving patients who may be difficult to communicate and work with.
8. Rating qualities that enable health professionals to give respectful care to patients	To stimulate engagement and discussion of particular styles of reacting in interactions with others and their implications for giving patients continuous respectful care.
9. Rating events that may affect teamwork and respect	To stimulate engagement with consideration of events or conditions (stressors) that might affect respect and teamwork.
10. Seeing another health professional being disrespectful	For the group to consider a) how a health professional would feel, and what they could say and do if they saw a colleague treating a patient with disrespect; b) what kind of interventions may be effective in drawing attention to and modifying disrespectful care.
11. Sharing our stories	To offer team members a chance to reflect, and to increase trust and mutual support among team members by sharing stories of success, humour and failure at work in an atmosphere of support.
12. Considering relevance of formative research findings to participants’ home facilities	For the teams to engage with research done by the STAR researchers and reflect on the applicability of general findings to their health facility.
13. Caring for yourself as a health professional in a stressful, busy environment	For the team to consider an account of disrespectful care from the qualitative formative research and suggest strategies that the midwife could have used to care for herself and maintain respectful care for her patients.
14. Advocacy for patients	For the team to consider an example of civil society advocacy related to antiretroviral accessibility and to consider ways in which they could act as advocates for their patients.

### Critical readers, reference group and piloting to assess acceptability

Once the initial draft of the toolkit was developed the activities were sent to two critical readers, one a professional nurse who was also an adult education specialist and trainer and the other a senior midwife and lecturer at a nursing college, both women, to read the toolkit and provide inputs regarding the academic level of the text and suitability of the exercises for district hospital maternity unit teams. The revised toolkit was then presented to a reference group comprising the maternity unit manager, midwives, nurses and a doctor at one of the participating hospitals. This group engaged with a sample of activities and provided helpful feedback on the clarity of language, activity instructions and time allocation for planned activities. Our discussion with this group also raised our awareness of a number of concerns that maternity unit staff would be likely to have about the STAR project, such as who the champions would be, what effect the project would have on their workloads, and whether they would be required to travel. These issues were subsequently included in the presentation when the intervention was introduced at each hospital.

A second revision of the toolkit was then piloted with maternity teams in two community health centres that were not part of the intervention study. Piloting focused on determining feasibility of the activities in terms of time requirement, ease of understanding the aims and how to facilitate the activity, as well as whether the activity enabled the desired level of participant interaction and engagement. Based on these two pilots, earlier inputs from the critical readers and the reference group, several changes were made to the text describing activities to improve clarity, to the layout of activities and activities were added, e.g., activity 13 around advocacy.

### Formation of STAR teams

The maternity unit in a district hospital comprises antenatal, postnatal, labour and neonatal units, which are managed as a single entity overseen by the maternity unit manager. At each participating district hospital, the STAR development team gave a presentation to introduce the project and answer any questions. Following this presentation, health professionals (nurses, doctors, unit managers) and allied health professionals (social workers, quality assurance managers etc.) involved in providing care across all four wards were invited to form one STAR team and two members from each facility either volunteered or were nominated by management to be STAR champions. The STAR team was asked to consider the following preferred characteristics in determining who would serve as champions: being able to prioritise time to facilitate learning sessions within their hospital, being passionate about making a difference in maternal and newborn care and keen to develop facilitation and training skills, clinical experience in maternal and newborn care, able to communicate well with district and facility personnel and the ability to attend the champions training workshop and follow-up support meetings. The teams were informed that being a STAR team member or champion was entirely voluntary and would not be remunerated. Anticipated risks from participation for the champions included the additional stress and burden of leading the team and taking responsibility for the STAR intervention on top of their existing role within the facility; possible risks for team members included that staff may choose to attend learning sessions in their off-duty time. Benefits were described as creating greater maternity unit team cohesion, learning facilitation skills and feeling a sense of pride and accomplishment when women have a positive birth experience. Earning CPD points was also a benefit for doctors in the teams.

### Champion training

A champion training workshop was held at a central venue in each of the study districts, bringing together two champions from each of the nine participating hospitals, some unit managers and district management representatives. The training was designed to last two and a half days. It was facilitated by the STAR development team including two adult education specialists from local universities (University of KwaZulu-Natal and Durban University of Technology), an organisational psychologist from the University of KwaZulu-Natal and a facilitator with experience in facilitating public health interventions. The training focused on orientation to the STAR intervention and toolkit, adult learning principles, group facilitation skills, how to establish a safe and respectful learning environment and completing monitoring and evaluation tools to document the change projects that each team would embark on. Facilitators also modelled how to facilitate the activities. Each champion and deputy champion had the opportunity to facilitate an activity and receive feedback during the training.

### STAR learning sessions

Following the training the champions returned to their facilities to launch learning sessions with their STAR teams. Each team was provided with a toolbox consisting of: stationery; flip charts; M&E forms; a memory stick with training videos; infographic posters summarising the intervention to display on the walls of the units; a modem with preloaded data for communication with the study mentors and for accessing additional videos recommended in the toolkit; and some project branding items like T-shirts and mugs for the STAR team to help raise awareness about the project within the facility and to encourage maternity unit staff to enjoy refreshments together during their learning sessions.

The M&E forms consisted of a form to record the names and contact details of team members and champions, a learning session record form (to record who participated in the learning session, activities undertaken, any challenges experienced and the date and preparation required for the next session), a form to plan change projects and a change project evaluation form. Exemplars of these forms were provided to guide champions.

The intention was that each team would meet monthly for about an hour, at a suitable time that caused minimal work disruption and when members from both day and night shift could participate. It was suggested that STAR learning sessions coincide with existing in-service learning activities where possible. After the first four or five sessions of getting to know each other and completing some of the preliminary participatory activities designed to stimulate awareness about respectful care, teams were encouraged to decide on a few priorities for change projects to improve respectful care and maternity unit teamwork. Tools were provided to assist teams with planning their projects to suggest ways to develop indicators for monitoring changes. Learning sessions were accredited with the Health Professions Council of South Africa for CPD points so health professionals who are required to obtain a minimum number of CPD points per year, which at the time of the study only applied to doctors, are able to accrue CPD points by attending STAR learning sessions. CPD points were awarded for participation in the champion training (12 points), 1 clinical point was awarded for each one-hour learning session attended and 12 points each for the mid-way and end of intervention workshop.

### Mentorship support

It was agreed that champions would receive on-site and telephonic support from the STAR facilitators approximately once every 4–6 weeks with support provided to plan and undertake STAR meetings until champions felt confident running group meetings alone. In addition, the mentorship included a WhatsApp group for each district consisting of the champions, study facilitators and district maternal and newborn managers. The purpose of these groups was for teams to share their experiences and progress with each other, for facilitators to provide ongoing encouragement and for district management to be aware of the activities of the STAR teams. A midway workshop in each study district was held to bring together the champion, deputy champion, an additional member of the STAR team and a maternity ward manager, together with the STAR development team, to share their experiences, identify challenges and successes, and receive additional support for the second period of the intervention. An end of intervention workshop bringing together both districts with provincial management was also planned to share experiences, celebrate change projects and to plan for the scale up to other districts and handover of ongoing mentorship for STAR teams to the district maternal, child and women’s health facilitators.

## Discussion

The experiences of developing the STAR intervention have resulted in important learnings on the process of developing health system interventions. These learnings have applicability to other public health interventions. The process involved in developing public health interventions often receives less attention and funding than the evaluation of such interventions [39,40] and has been referred to as the ‘black box’ of intervention research because important processes and decision-making in the stage of intervention development are seldom reported or published [40]. A scoping review of strategies to improve interpersonal communication in maternal and newborn care highlighted a lack of detail on the design of interventions, which reduces the opportunities for others to learn and adapt such strategies [41].

An important learning from the development of the STAR intervention was the need to allocate sufficient time and financial resources to the development process. For the STAR intervention the funding grant allowed for a full 12 months to develop the intervention which our experience indicates was necessary to complete all the steps. Piloting the toolkit activities was also an essential step to test feasibility on how health professionals understood, responded to and participated in the activities and enabled changes to be made prior to implementation. Hoddinott [40] in her editorial on intervention development studies argues that pilots generate data that can validate decisions and challenge critical assumptions quickly and at an early stage when developing an intervention.

A strength of the STAR intervention development was the intentional multi-disciplinary team approach led by a professor of adult education. Public health interventions are often developed by researchers with health backgrounds. However, for complex, multi-component interventions, engagement of interdisciplinary teams with diverse skill sets increases the likelihood that the intervention will be fit for purpose [39]. The STAR development team incorporated expertise in adult education and organisational psychology, in addition to team members with public health and medical expertise. This was critical to ensure that the training and toolkit activities took into account adult learning principles and techniques and considered issues of power and hierarchy through participatory approaches such as role-play’s that help to level the learning space when inter-professional teams are brought together. The initial stages of planning the intervention approach and toolkit activities took time as we needed to understand each other’s professional ‘languages’ and epistemologies and establish trust in the process through regular engagement and open feedback. This was reflected in how the team allocated tasks based on skills and experience and took accountability for the work produced. The STAR development team also met regularly to reflect on progress and address team dynamics to assess what was working and what wasn’t and used these engagements to address any issues and challenges.

A particular tension for the public health and biomedical members of the STAR development team was the need to cope with less control over the intervention than is typical of public health interventions. With the participatory, locally led approach we had to give control over to the maternity units in terms of the selection of champions and team members and the running of learning sessions. Typical public health interventions, particularly those being evaluated through a randomised-controlled trial design, involve high levels of control over the intervention implementation. In this participatory intervention our intention was for the hospitals to select their own STAR champions and we, therefore, had to be clear during our introductory visits to the facilities about the characteristics considered ideal for champions and then allow the selection process and project implementation to be locally led.

Another learning from the STAR intervention development process was the value in engaging early and at regular intervals with key provincial and district stakeholders and being cognizant of the political context in terms of policy changes. This enabled buy-in and the smooth launch of the intervention within participating hospitals due to stakeholder requirements having been taken into account in the development stage. The most recent United Kingdom Medical Research Council (UKMRC) framework for developing and evaluating complex interventions recommends that meaningful engagement with appropriate stakeholders in the development and delivery of complex interventions maximises the potential of developing an intervention that is likely to have positive impacts on health and to enhance prospects of achieving changes in policy or practice [42].

In terms of the political context, the timing of the intervention development coincided with the launch of new minimum standards for maternity care in KwaZulu-Natal province [43] which elevated the priority given to respectful maternity care. The first minimum standard is that all women should be allowed a companion of choice throughout labour. Companionship in labour has been recognised as an effective intervention to promote respectful maternity care [44]. The background to the new minimum standards outlines that the provincial health portfolio committee identified maternity services as being responsible for a large number of medical legal claims related to unsafe and disrespectful care [43]. The STAR intervention was therefore responding directly to a policy window to address a health care need that was also a political priority.

The participatory and self-directed approach to STAR meetings attempts to take into account the history and social drivers of disrespectful maternity care in South Africa. Disrespect within maternity care has been found to be driven by both social and structural factors, which include social norms, the political economy and health system policies [45]. In terms of social norms, South Africa has one of the highest rates of gender-based violence in the world [46], and the majority of nurses and midwives are women living in environments where they may themselves be experiencing gender-based violence and powerlessness [1,47]. The activities within the toolkit specifically challenge health professionals to reflect on their personal motivations for entering the profession and the barriers they face to providing respectful care, as well as considering situations from the perspective of women in their care as ways to challenge power divides and encourage empathic communication.

The social and economic context of South Africa is characterised by extremely high levels of inequality at multiple levels, including between urban and rural areas, the public and private health care system, racial groups as well as gender inequality [48]. From a health system perspective the lack of accountability mechanisms has enabled disrespectful maternity care to be normalised [45]. The STAR approach therefore aimed to give control to health professionals to identify their own priorities for improving their working environment and ways of relating to women in their care and encouraging team members to hold each other accountable, rather than imposing external solutions and further entrenching powerlessness.

Outcomes and impact will be measured through a mixed-method before-and-after evaluation. Cross-sectional surveys will assess the impact of the intervention on two primary outcomes, person-centered maternity care and hospital organisational culture. A qualitative process evaluation including descriptive case studies will document the experiences and process of STAR implementation. The evaluation will focus on processes, context, barriers and enablers. An economic evaluation will also document the health system costs of taking this intervention to scale within district hospital settings.

## Strengths and limitations

A possible limitation of the STAR intervention is that it relies on health professionals to be self-motivated and self-direct their team learning with the intention that a low level of mentorship support would be required from the STAR facilitators. This could result in the intervention not being prioritised by health professionals due to competing demands of clinical-focussed learning activities and staff shortages at some hospitals or that the level of external support increases, which has implications for sustainability of the approach. The intention was for ongoing mentorship, undertaken by STAR-supported facilitators for the research period, to be taken over by district maternal, child and women’s health facilitators on completion of the research.

A potential strength of the STAR approach is that it brings together multi-disciplinary teams to reflect and solve challenges together. This is particularly important for maternity units where cultures of blame between nurses and doctors or nurses and management are often normalised, particularly when adverse events occur, resulting in lack of trust amongst the maternity team [49].

## Conclusion

Grounded in a recognition of health systems as learning organisations and the need for sustainable learning and change processes to be locally driven, we brought together a multi-disciplinary team to design the STAR intervention using an iterative approach over one year involving multiple processes of formative research, expert review and piloting, all of which helped to create a tool kit and implementation approach. There is value in documenting the development of complex public health interventions to expose the ‘black box’ and to enable other researchers to share in the learnings.

## Supplementary Material

Supplementary file Intervention development paper.docx
