# Supplementary material for: Strengthening teamwork and respect (STAR) in maternity units: developing a health system intervention in South Africa
Source: Glob Health Action. 2025 Feb 3;18(1):2440982. doi: 10.1080/16549716.2024.2440982 (PMC11792138; doi:10.1080/16549716.2024.2440982)
Supplement: Supplementary file Intervention development paper.docx [file ZGHA_A_2440982_SM1366.docx]

**Supplementary file:**

Supplementary figure 1: Schematic diagram of formative research

**Quantitative survey** **with mothers** who had recently delivered in participating health facilities

Person-centred maternity care (PCMC) tool

Interviews conducted in primary health care clinics 6-10 weeks post-partum

N= 908 mothers

Exploring mother’s experiences of maternity care

Formative research to inform development of the STAR intervention

**Qualitative study with health professionals** working in maternity units in 11 participating hospitals

35 In depth interviews conducted with health professionals including health managers doctors, midwives and nursing assistants

**Quantitative survey with health professionals** working in maternity units in 11 participating health facilities

Dimensions of learning organization Questionnaire (DLOQ)

N= 116 health professionals

**Qualitative study** **conducted with mothers** who had recently delivered at 5 participating health facilities

10 Focus Group Discussions (FGDs) conducted comprising:

5 FGDs conducted with mothers whose infants were admitted in the neonatal unit conducted within hospitals

5 FGDs with mothers in the community conducted at community-based venues

Exploring health professionals’ perspectives about provision of maternity care

Example of a vignette case study from the STAR toolkit used in the introduction to the concept of respectful care

Example of a case study from activity 13

Example of artwork from activity 6 in the STAR toolkit

Link to the STAR animation video

<https://drive.google.com/file/d/19l2B26cW3e8kPUC8xsqKmu4kxBnOJng5/view>
